# A Two-Step Simulated Annealing Algorithm for Spectral Data Feature Extraction

**DOI:** 10.3390/s23020893

**Published:** 2023-01-12

**Authors:** Jian Pei, Liang Xu, Yitong Huang, Qingbin Jiao, Mingyu Yang, Ding Ma, Sijia Jiang, Hui Li, Yuhang Li, Siqi Liu, Wei Zhang, Jiahang Zhang, Xin Tan

**Affiliations:** 1 Changchun Institute of Optics, Fine Mechanics and Physics, Chinese Academy of Sciences, Beijing 100049, China; 2 University of Chinese Academy of Sciences, Beijing 100049, China; 3 College of Computer Science and Technology, Jilin University, Changchun 130012, China; 4 Center of Materials Science and Optoelectronics Engineering, Chinese Academy of Sciences, Beijing 100049, China

**Keywords:** spectral detection, feature extraction, cyanobacteria biomass, lake eutrophication, quantitative inversion

## Abstract

To address the shortcomings in many traditional spectral feature extraction algorithms in practical application of low modeling accuracy and poor stability, this paper introduces the “Boruta algorithm-based local optimization process“ based on the traditional simulated annealing algorithm and proposes the “two-step simulated annealing algorithm (TSSA)”. This algorithm combines global optimization and local optimization. The Boruta algorithm ensures that the feature extraction results are all strongly correlated with the dependent variable, reducing data redundancy. The accuracy and stability of the algorithm model are significantly improved. The experimental results show that compared with the traditional feature extraction method, the accuracy indexes of the inversion model established by using the TSSA algorithm for feature extraction were significantly improved, with the determination coefficient R^2^ of 0.9654, the root mean square error (RMSE) of 3.6723 μg/L, and the mean absolute error (MAE) of 3.1461 μg/L.

## 1. Introduction

Spectroscopy has received much attention in the last two decades and been used in many fields, such as food, pharmaceuticals, chemical analysis, environmental monitoring, and precision agriculture [1]. 

However, high-resolution spectral data overlap with extensive data redundancy and contain many bands of information unrelated to the measured components, adversely affecting the modeling effect. Thus, accurately extracting valuable features from the “high-dimensional” spectral information for modeling has become particularly important and is an urgent problem to be optimized and solved in this field of research. 

The simulated annealing (SA) algorithm was first developed by N. Metropolis et al. in 1953 [2]. In 1983, S. Kirkpatrick [3,4] and others successfully introduced the idea of annealing into combinatorial optimization. It is a stochastic global-search combinatorial optimization method with the feature of fast global optimization. The SA algorithm considers spectral characteristics from the global view of data and extracts features in an optimized combination. It avoids overfitting the model training owing to less feature information. It does not transform the original data information, thus ensuring the correct interpretation of the meaning of the data information. However, the SA algorithm tends to have slow convergence when solving larger-scale combinatorial optimization practical problems. Moreover, the information in the optimization results is often unrelated to or has a weak correlation with the dependent variable. Many scholars have combined their research areas to improve the SA algorithm. Xiutang Geng [5] et al. used three different probability combinations based on the standard SA algorithm in the search process. They then used greedy search techniques to speed up the algorithm’s convergence. Dimitris N. Simopoulos [6] et al. combined the SA algorithm with a dynamic economic scheduling method. They proposed new rules concerning the adjustment of the control parameters of the SA algorithm. Bernardo Morales-Castañeda [7] et al. modified the original SA incorporating two new operators, folding and reheating, to improve its search capability. Eduardo Rodriguez-Tello [8] et al. proposed a SA algorithm for solving the graph bandwidth minimization problem. The algorithm is based on three salient features, including the original internal representation of the solution, a highly discriminative evaluation function, and an effective neighborhood. When the SA algorithm is used for spectral feature extraction, the algorithm still has slow convergence and poor stability, resulting in low modeling accuracy. Still, there are a few reports of optimization improvements for its use in spectral feature extraction. 

The SA algorithm uses random substitution in the global optimization process to update the spectral feature combinations, which has a high probability of causing unrelated or weakly correlated information with the dependent variable to enter the feature combinations. The SA algorithm itself cannot discriminate correlations. Moreover, the algorithm relies only on global optimization, which can lead to the problem of jumping out of important bands too quickly when updating combinations, leading to poor modeling results.

The Boruta algorithm is a wrapper method built around a random forest classifier and is widely used in feature extraction [9]. Shaheen et al. used geostatistical methods to assess the spatial distribution of the heavy metals, chromium (Cr), cadmium (Cd), and lead (Pb), at three soil depths and used the random forest (RF) function of the Boruta algorithm to select the significant contributing variables to soil contamination [10]. Subbiah S et al. overcame the problem of poor accuracy and misclassification of network intrusion detection by the Boruta feature selection and grid search random forest (BFS-GSRF) algorithm [11]. Kent B et al. used the Boruta algorithm to successfully find the impedance spectrum characteristics of the studied biological tissue [12]. Modeling accuracy cannot be relied upon as the sole criterion for judging the importance of features. The decrease in modeling accuracy after removing features is enough to show the importance of features, but the lack of this effect is not enough to show that it is not essential. The Boruta algorithm determines the variable correlation by comparing the correlation between real features and shaded features. It iteratively removes irrelevant information that is proven by statistical validation and can select all sets of features relevant to the dependent variable, helping the algorithmic model more fully understand the factors influencing the dependent variable. Therefore, the Boruta algorithm was chosen to determine the importance of feature information in this study.

To address the above problems, this paper introduces the “Boruta algorithm-based local optimization process” based on the traditional simulated annealing algorithm. It proposes the two-step simulated annealing algorithm (TSSA) for spectral feature extraction. The TSSA algorithm combines global optimization and local optimization to improve modeling accuracy while ensuring that the search results are free of weakly correlated bands, reducing the redundancy of extracted features and improving model stability.

The organization of the paper is as follows.

Section 1 is an introduction. This chapter introduces the main factors that currently limit the detection accuracy of high-resolution spectral data, describes the simulated annealing algorithm’s advantages and shortcomings, and introduces this paper’s research content and research methods in response to these problems. Section 2 first analyzes the main reasons for the poor performance of the traditional simulated annealing algorithm in practice and proposes the TSSA algorithm for these main reasons. The principle of the TSSA algorithm, the structure of the algorithm, and the steps of how the algorithm processes data are detailed. Section 3 describes the dataset source and acquisition. First, the experimental conditions and experimental apparatus are described. Then, the detailed operational steps for collecting spectral data and the chemical assay for chlorophyll a (Chla, μg/L) concentration of the samples are described. Section 4 introduces the data processing approach and operating procedures to establish a quantitative inversion algorithm for cyanobacterial Chla in eutrophic shallow-water lakes and discusses the modeling accuracy. Six approaches are used in the feature extraction section. The experimental results are discussed to demonstrate the superiority of the TSSA algorithm proposed in this paper. Section 5 summarizes the research content of this paper and shares the future outlook.

## 2. Two-Step Simulated Annealing Algorithm

According to the characteristics of high-dimensional spectral data, there are two main reasons for the unsatisfactory effect of the SA algorithm in spectral feature extraction: (1) The presence of information in the spectral band combination is unrelated or weakly correlated with the dependent variable; (2) The algorithm is globally optimized to jump out of important information too quickly when randomly updating the wave combinations. To address the above problems, this paper introduces a Boruta algorithm-based local optimization process based on the traditional SA algorithm. The new algorithm is called the two-step simulated annealing algorithm (TSSA).

**Figure 1:**
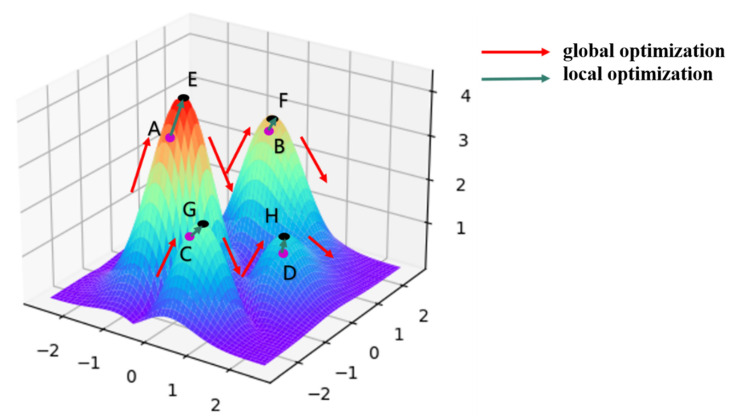
TSSA algorithm optimization process.

As shown in Figure 1, the optimization process of the TSSA algorithm is analogous to the optimization process with the aim of climbing to the highest point (E) of a mountain peak. Owing to the two problems mentioned earlier, the SA algorithm does not reach the top of the peak during the global optimization search but jumps out of the section after reaching points A, B, C, and D under the conventional initial parameter settings. Point B is the optimal solution filtered on this basis, which still has a large gap with objective (E). TSSA’s unique “Boruta algorithm-based local optimization process” can quickly complete the local optimization operations from A to E, B to F, C to G, and D to H and find the highest point (E) using less time and computational resources.

### 2.1. The Global Optimization Search Process for Simulated Annealing

The TSSA algorithm has a structure with a memory function. Let the initial band combination of the algorithm be *x* = {w_1_, w_2_, ..., w_n_} and the optimal band combination be *x_b_*. Then, initialize *x_b_* = *x*. The first step of the algorithm is the global optimization process. At this point, for *x* randomly replacing *m* non-coincident bands to form *x’*, the value of *m* is determined by Equation (1):

(1) m=⌈|N(0,0.1×n×T)|⌉^⌈0.1×n⌉

where *T* is the temperature at the current moment of the algorithm and *n* is the number of bands in the combination.

Statistical mechanics studies have shown that the probability of a particle staying in the state *i* at temperature *T* satisfies the Boltzmann distribution:

(2) $$P_{T}\left(x=i\right)=\frac{1}{Z\left(T\right)}exp\left|-\frac{E\left(i\right)}{k_{b}T}\right|$$

(3) $$Z\left(T\right)=\sum_{j\in S}exp\left|-\frac{E\left(j\right)}{k_{b}T}\right|$$

where *E*(*i*) is the energy of state *I*; *k_b_* > 0 is the Boltzmann constant; *T* is the temperature; and *x* denotes the random variable of the current state of the material. *Z*(*T*) is the normalization factor of the probability distribution, and *S* represents the set of state spaces.

In the high-temperature state, there is obviously the following:

(4) $$\underset{T\to \infty }{\lim}\frac{exp\left|-\frac{E\left(i\right)}{k_{b}T}\right|}{\sum_{j\in S}exp\left|-\frac{E\left(j\right)}{k_{b}T}\right|}=\frac{1}{\left|S\right|}$$

where |*S*| denotes the number of states in the set *S*. This indicates that all states have the same probability at high temperatures.

Assuming that the current annealing temperature is *T*, whether or not to accept the update of *x* = *x*′ depends on the probability calculated by Equation (5). This is the famous Metropolis criterion, which is the core idea of the SA algorithm:

(5) $$P\left(x\to x^{\prime }\right)=\{\begin{array}{c} \;\;\;\;\;\;\;\;\;\;1\;\;\;\;\;\;\;\;\;\;\;\;,\;f\left(x^{\prime }\right)>f\left(x\right)\\ e^{\frac{f\left(x^{\prime }\right)-f\left(x\right)}{T}}\;,otherwise \end{array}$$

### 2.2. Boruta Algorithm-Based Local Optimization Process

The degree to which the bands in x contribute to improving the modeling effect during the algorithm annealing search varies, and information unrelated or weakly correlated with the dependent variable adversely affects the results. Replacing it in the global optimization process causes the algorithm to fall into a local optimum, which violates the core idea of the SA algorithm. For this purpose, the algorithm introduces the Boruta algorithm-based local optimization process.

When the global optimization process in the first step satisfies the condition *f*(*x*′) > *f*(*x_b_*), the algorithm enters the second step: the Boruta algorithm-based local optimization process. The process first uses the Boruta algorithm to filter out s irrelevant and weakly correlated bands in *x*′ and then replaces them with s randomly selected, not coinciding bands to obtain *x_p_*.

In the SA process, when the temperature decreases,

(6) $$\underset{T\to 0}{\lim}\frac{exp\left(\frac{E\left(i\right)-E_{min}}{k_{b}T}\right)}{\sum_{j\in S}exp\left(\frac{E\left(j\right)-E_{min}}{k_{b}T}\right)}\;=\underset{T\to 0}{\lim}\begin{array}{c} \frac{\exp\left(\frac{E\left(i\right)-E_{min}}{k_{b}T}\right)}{\sum_{j\in S_{min}}\exp\left(\frac{E\left(j\right)-E_{min}}{k_{b}T}\right)-\sum_{j\in S_{min}}\exp\left(\frac{E\left(j\right)-E_{min}}{k_{b}T}\right)} \end{array}\;=\;\;\;\;\;\;\;\;\underset{T\in 0}{\lim}\frac{exp\left(\frac{E\left(i\right)-E_{min}}{k_{b}T}\right)}{\sum_{j\in S_{min}}exp\left(\frac{E\left(j\right)-E_{min}}{k_{b}T}\right)}\;=\{\begin{array}{c} \frac{1}{\left|S_{min}\right|}\;\;\;\;\;\;\;\;\;\;\;\;\;\;\;\;\;\;\;\;\;\;if\;\;i\in S_{min}\;\\ \;\;\;\;\;\;\;\;\;\;\;0\;\;\;\;\;\;\;\;\;\;\;\;\;\;\;\;\;\;\;\;\;\;\;\;\;\;otherwise\;\;\;\;\;\;\;\;\;\; \end{array}$$

where $E_{min}=\begin{array}{c} min\\ j\in S \end{array}E\left(j\right)$, and $S_{min}=\left\{i|E\left(i\right)=E_{min}\right\}$.

The above equation shows that when the temperature drops to a deficient level, the material enters the minimum energy state with a high probability. This algorithm plunges the temperature to 0 degrees directly in the second step and uses Equation (7) as the basis for determining whether to accept the *x_b_* = *x_p_* update:

(7) $$P\left(x_{b}\to x_{p}\right)=\{\begin{array}{c} \;\;\;\;\;\;\;\;\;\;\;\;1\;\;\;\;\;\;\;\;\;\;\;\;,\;f\left(x_{p}\right)>f\left(x_{b}\right)\\ \;\;\;\;0\;\;\;\;\;\;\;\;\;\;\;\;,\;otherwise \end{array}$$

At this time, the material enters the minimum energy state, the algorithm follows the principle of local optimization, and after iterating this process L_p_ times, the algorithm returns to the first step of the global optimization process until the temperature T has dropped to less than the termination temperature T_min_, the algorithm operation ends, and x_b_ is the final optimization result.

**Figure 2:**
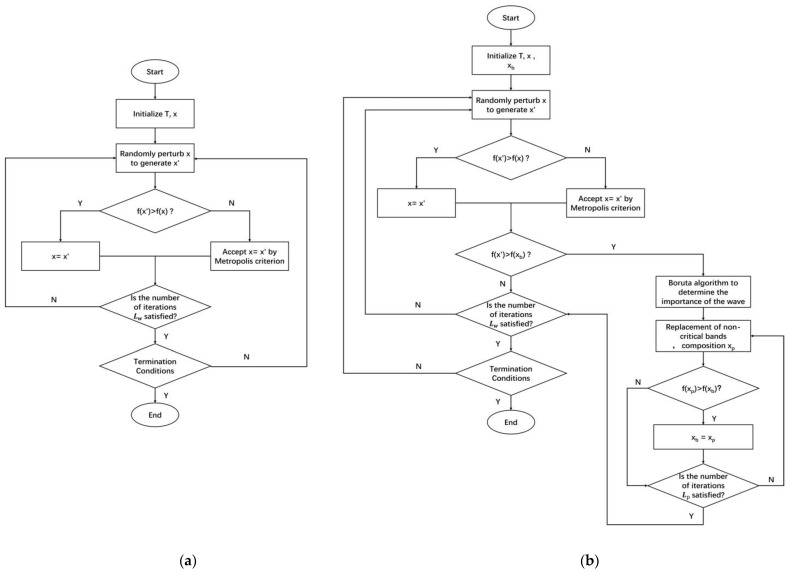
Algorithm flow chart (**a**) Original SA (**b**) TSSA.

In Figure 2a,b the flow chart of simulated annealing and TSSA algorithms is shown, respectively. The TSSA algorithm has the same termination conditions as the SA algorithm. The algorithm terminates when the temperature T is less than the termination temperature T_min_.

The objective function of the algorithm is defined as *f*(*x*):

(8) $$\;f\left(x\right)=\frac{1}{1+t\left(x\right)}$$

where *t*(*x*) is the mean square error (MSE) of the prediction results obtained by cross-validating the training set data using the bands in *x*. The larger the value of the objective function *f*(*x*), the better:

(9) $$t\left(x\right)=\frac{1}{n}\overset{n}{\underset{i=1}{\sum }}{\left(y_{predict}-y_{true}\right)}^{2}$$

## 3. Study Area and Data Measurement

Lakes are rich natural systems. Over the decades, many lakes have experienced increasing eutrophication and water-quality degradation [13,14,15,16,17]. Adequate algal biomass and suitable environmental conditions are essential for forming cyanobacterial blooms. Chla is a common indicator of algal biomass in lake science and ocean color remote-sensing science.

### 3.1. Study Area

Chaohu Lake is the fifth largest freshwater lake in China, located in central Anhui Province, with an area of 770 km^2^ (31°25′–31°43′ N, 117°17′–117°51′ E) and an average water depth of 3.0 m [18]. Taihu Lake (30°56′–31°34′ N, 119°54′–120°36′ E), located in the middle and lower reaches of the Yangtze River, south of Jiangsu Province, China, is the third largest freshwater lake in China, with a surface area of 2338 km^2^ [17] and an average water depth of 2.1 m. Water pollution and eutrophication in Chaohu and Taihu lakes have become increasingly serious over the past three decades [19,20,21,22], threatening their use as drinking water sources.

### 3.2. Data Collection

**Figure 3:**
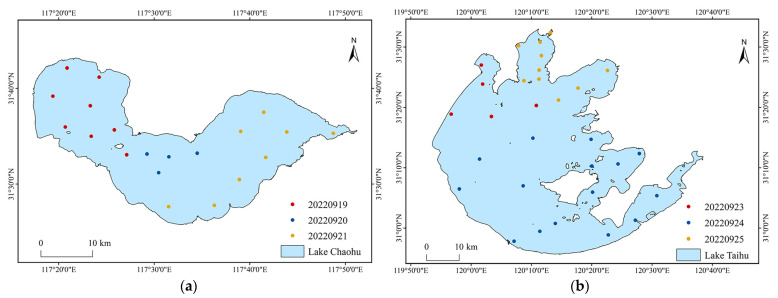
Study area and sampling locations (**a**) Chaohu (**b**) Taihu.

**Figure 4:**
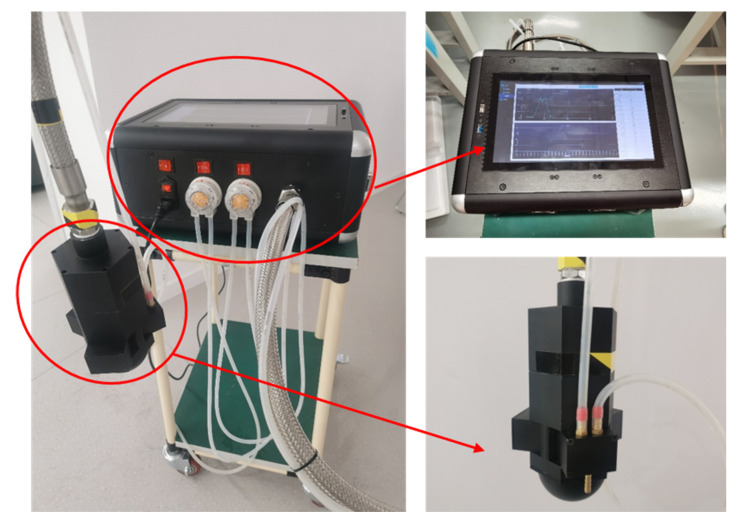
Lake cyanobacterial biomass spectral detection system.

**Figure 5:**
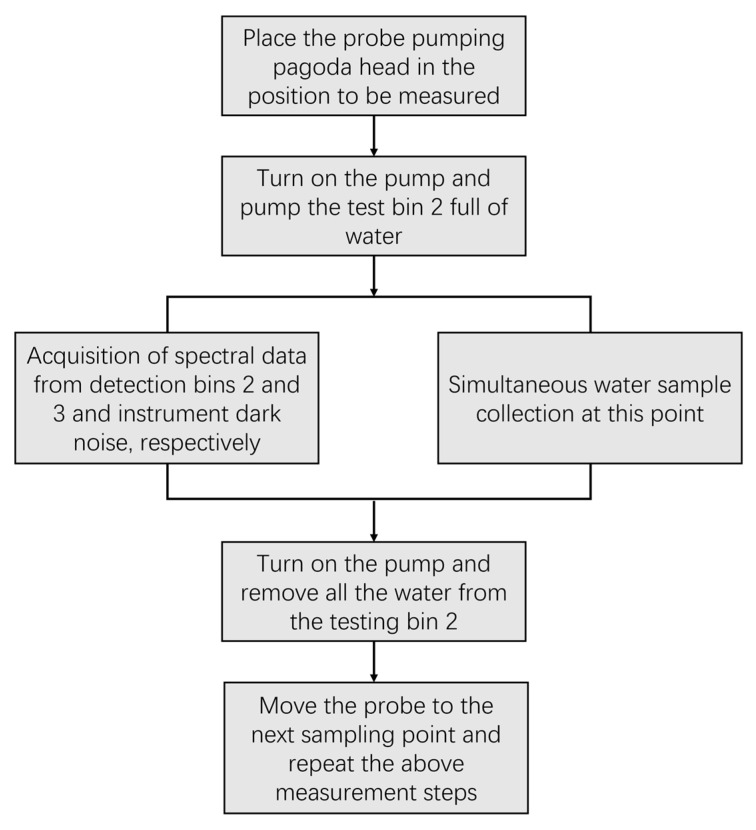
Data collection flow.

Field survey measurements were conducted in Chaohu Lake (19 September to 21 September) and Taihu Lake (23 September to 25 September) in 2022, with 20 and 30 monitoring points, respectively. In Figure 3a,b, the geographic coordinates of the sampling sites in Chaohu and Taihu Lake are shown. In the range of 0–2 m underwater, the sample data of one–three depths were taken for each sampling point. The self-developed lake cyanobacterial biomass spectral-detection system was used to measure the spectral data of water bodies. The main principle of this equipment is to collect the absorption spectrum of substances in the water body through the alignment layout of the light source and the spectrometer, designed with a separate structure above and below the water to meet the needs of underwater operations from 0 to 2 m. The lake cyanobacterial biomass spectral detection system is shown in Figure 4. The underwater probe section sets up three test bins. The No. 1 test bin is empty, the No. 2 test bin load has lake water, and the No. 3 test bin load has distilled water prepared by the laboratory. The signal-to-noise ratio is greater than 2000:1. The spectral range of the equipment covers 200–1000 nm; the spectral resolution is better than 1 nm. When collecting spectral data, we place the probe pumping pagoda head in the position to be measured. After detection bin 2 has been pumped full of water, the spectral data of detection bins 2 and 3 and the spectrometer dark noise data are collected, respectively. After the collection has completed, detection bin 2 is controlled for drainage. When the detection bin 1 and 2 spectral curves consistently prove that all the water has been drained, the probe position is moved for the subsequent sampling-point measurement. The data collection flow is shown in Figure 5. The water samples were collected simultaneously using a water collector, stored at a low temperature (4 °C) under light-proof conditions, and then filtered within 6 h after the collection had been completed. Chla concentrations of water samples were measured in the laboratory at the end of the collection survey (no more than 5 days).

Chla concentrations of water samples were measured in the laboratory. The water samples were filtered with Whatman GF/C glass fiber filters (pore size of 1.1 μm), and pigments were extracted using 90% acetone. Chla values were calculated using absorbance measured at 630, 645, 663, and 750 nm with a Shimadzu UV-2600 spectrophotometer.

## 4. Results and Discussion

### 4.1. Data Preprocessing

**Figure 6:**
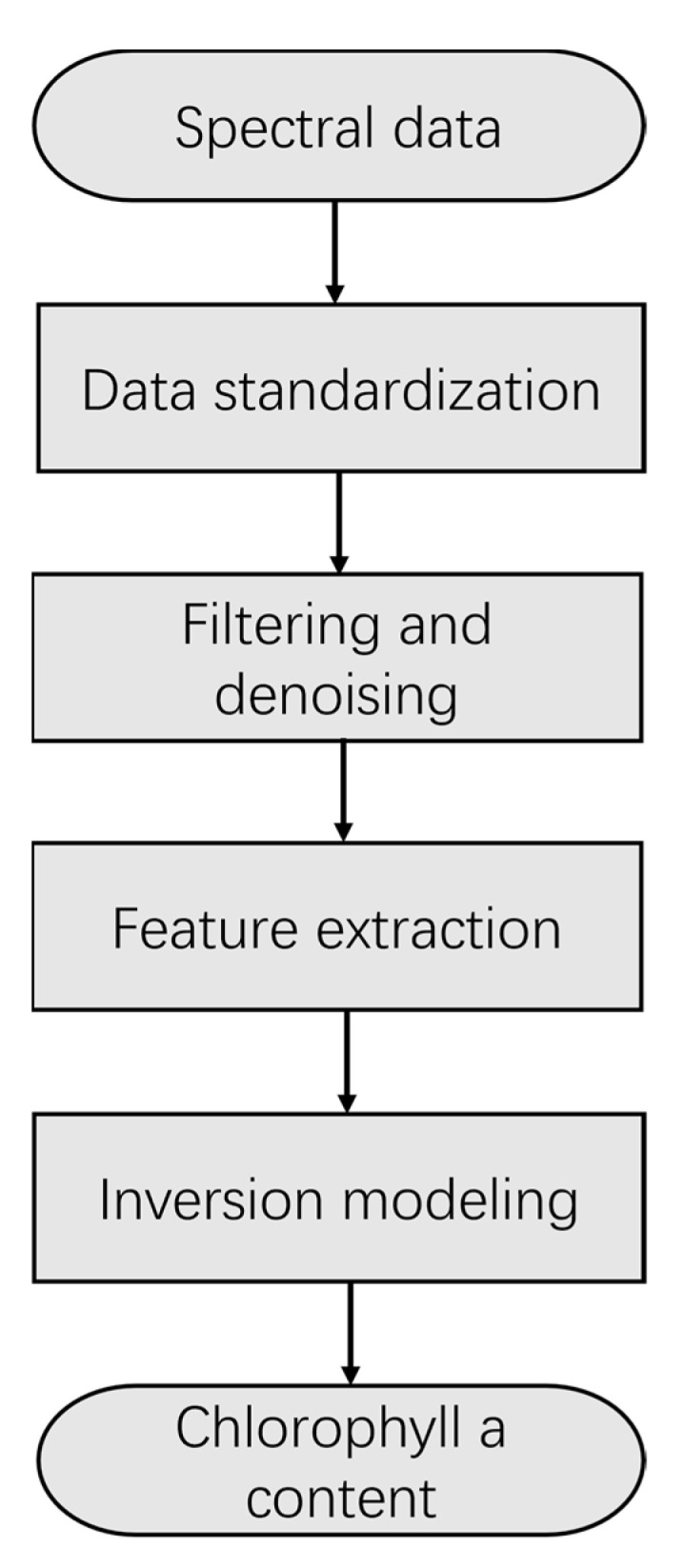
Data processing flow.

In this paper, the spectral data collected from field experiments in Chaohu Lake and Taihu Lake were analyzed together with the results of water sample assays to construct an algorithmic model for the inversion of cyanobacterial Chla in the water column of eutrophic shallow lakes. The data processing mainly included four steps: data normalization, filtering and denoising, feature extraction, and inversion modeling. The data processing flow is shown in Figure 6.

The spectral range of the device was 200–1000 nm, covering UV–visible–-near infrared waves. The water absorbs significantly in the near-infrared band, so the spectral data of detection bin 3 were used as a standard to make a difference in the spectrum with the spectral data of detection bin 2. The absorption spectrum was calculated using Equation (10) for calibration, and the SG filter is used for denoising:

(10) $$absorption\;spectrum=lg\left(I_{3}\right)-lg\left(I_{2}\right)$$

In the formula, *I_3_* is the spectral data of the distilled water in the No. 3 detection bin, and *I_2_* is the spectral data of the lake water in the No. 2 detection bin.

The spectral differentiation operation was performed by numerically simulating the original spectrum and calculating the differentiation values of different orders to obtain the inflection points of the differentiated spectrum as well as the maximum and minimum values and wavelength positions of the absorption spectrum. The spectral differentiation process can eliminate some noise effects in the original spectrum. Continuum removal is a common method of spectral feature-enhancement processing. It normalizes the spectral reflectance to 0 to 1, highlighting the absorption and reflection features of the spectrum and normalizing them to a uniform spectral background, which facilitates feature comparison with the spectral curves of other sampling points.

**Figure 7:**
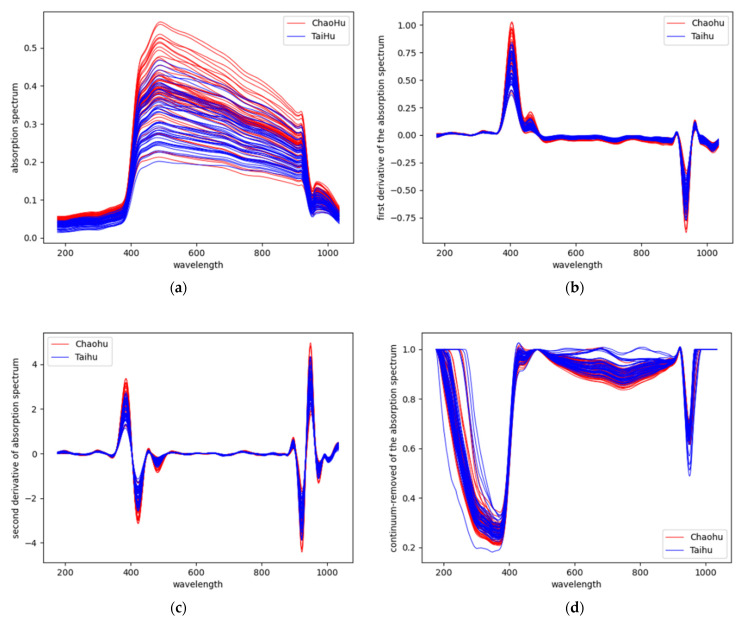
Spectral curves of test data (**a**) Original absorption spectrum (**b**) Original absorption spectrum were processed using first-order differentiation (**c**) Original absorption spectrum were processed using second-order differentiation (**d**) Original absorption spectrum were processed using continuum removal.

Figure 7a shows the spectral absorption curve of the water sample calculated using Equation (10). The calibrated data were processed using first-order differentiation, second-order differentiation, and continuum removal to highlight features that were difficult to represent in the original spectra. The data obtained after first- and second-order differentiation of the spectral curves in Figure 7a are too small in order of magnitude. The spectral curves shown in Figure 7b,c are the results obtained by subjecting the spectral curves in Figure 7a to first-order and second-order differentiation, respectively, and then enlarging them by a factor of 100 in equal proportion. As Figure 7d shows, the spectral curve is obtained after continuum removal processing of the spectral curve in Figure 7a.

### 4.2. Feature Extraction

A total of 107 data samples were collected from the field experiments conducted at Chaohu Lake and Taihu Lake, which were randomly divided into a training set and validation with 79 data samples and 28 data samples, respectively. Feature extraction of the normalized spectral data was performed using the correlation coefficient method, band ratio method, principal component analysis (PCA), successive projections algorithm (SPA), SA algorithm, and TSSA. The SA algorithm had an initial temperature (T_max_) of 200, an end temperature (T_min_) of 1, a temperature decay coefficient (k) of 0.95, and a maximum Markov chain length (L_w_) of 50. The TSSA algorithm had the parameters T_max_ as 100, T_min_ as 1, k as 0.95, L_w_ as 50, and the number of local optimization iterations as L_p_ as 1000. The number of bands n in the optimized combination of SA and TSSA algorithms was taken from 2 to 30 to calculate, and the value of n when the objective function was optimal was its best value.

### 4.3. Model Building and Accuracy Comparison

Inputting the extracted feature bands into a back-propagation (BP) neural network to model a quantitative inversion algorithm for cyanobacterial Chla in eutrophic shallow water lakes. A three-layer BP neural network was used for training. The number of neurons in the hidden layer was 100, the activation function was sigmoid, and L1 regularization was used. The calculation results show that the best modeling results were obtained by using the data obtained from the first-order differentiation of the original absorption spectrum among the four normalization methods. The spectral feature bands obtained using the six feature extraction methods are shown in Table 1, and the modeling accuracy comparison is shown in Table 2.

**Figure 8:**
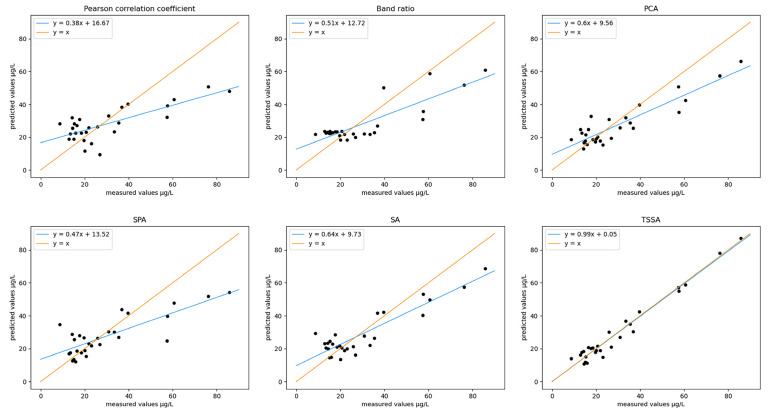
Comparisons of the measured and predicted values of the validation dataset using the results obtained from the six feature extraction methods for modeling predictions.

Compared with the other five feature extraction methods, the TSSA algorithm has obvious superiority in spectral feature extraction. First, in principle, the “correlation coefficient method” and the “band ratio method” only rely on a small amount of band information to establish a linear fit to the calibration data. The models are easily overfitted in the training set and poorly predicted in the validation set [23,24]. “Principal component analysis” (PCA) is suitable for data with a strong correlation between variables, but if the correlation of the original data is weak, it will not play a good role in dimensionality reduction, and the meaning of principal components will be more ambiguous than that of the original data [25,26]. The “Successive projections algorithm” (SPA) does not consider the maximization of irrelevance globally, only locally [27,28]. The SA algorithm has only a global optimization process and jumps out of important bands too quickly when updating combinations, leading to poor modeling results. Moreover, the algorithm cannot determine feature relevance [2,3,4]. The TSSA algorithm has both global and local optimization, which can ensure the efficient operation of the algorithm and does not transform the original data. It has a clear interpretation of the meaning of the feature extraction results. The combined optimized feature extraction avoids the overfitting caused by fewer features. The addition of the Boruta algorithm enables it to accurately determine the correlation between spectral features and dependent variables. It can eliminate irrelevant variable interference and reduce data redundancy. Second, in terms of modeling accuracy comparison results, as shown in Table 2, compared with the other five feature extraction methods, using the TSSA algorithm for feature extraction, the modeling accuracy indexes are significantly improved, with the determination coefficient R2 increased by 0.1908–0.4623, RMSE decreased by 5.7039–10.249 μg/L, and MAE decreased by 4.1575–7.6202 μg/L. As the comparison in Figure 8 shows, the fitted straight line between the predicted and measured values of the validation dataset using the TSSA algorithm is y = 0.99x + 0.05, which has the highest overlap with y = x, indicating the best modeling effect.

## 5. Conclusions

This paper investigated the performance optimization and application of the SA algorithm. The Boruta algorithm-based local optimization process was introduced based on the traditional SA algorithm, and the “two-step simulated annealing algorithm” (TSSA) was proposed. This algorithm combines global optimization and local optimization. The algorithm removes irrelevant and weakly correlated information from the features, reduces the redundancy of feature extraction, and effectively improves the modeling accuracy. Data were collected using self-developed spectral detection equipment for field measurements in the typical eutrophic shallow lakes of Chaohu and Taihu. The experimental results show that compared with the traditional feature extraction method, the accuracy indexes of the inversion model established by using the TSSA algorithm for feature extraction were significantly improved, with the determination coefficient R2 increased by 0.1908–0.4623, RMSE decreased by 5.7039–10.249 μg/L, and MAE decreased by 4.1575–7.6202 μg/L. This study provides new ideas and research directions for spectral feature extraction research and lays the foundation for accurate, rapid, and low-cost detection of Chla content in cyanobacteria using spectroscopic techniques.

## Figures and Tables

**Table 1:** Feature bands obtained using the six feature extraction methods.

Feature Extraction Method	Spectral Characteristic Band
Correlation coefficient method	905.04 nm
Band ratio method	987.47/674.38 nm
PCA	Six principal components
SPA	660.56, 364.67, 973.41, 357.53 nm
SA	516.69, 557.17, 993.93, 970.30, 816.23, 877.13, 677.72, 357.53, 666.85, 446.62, 994.61, 717.34, 927.75, 798.38 nm
TSSA	992.57, 976.85, 686.87, 727.11, 463.64,
	352.76, 678.97, 639.01, 959.88, 530.55, 970.30 nm

**Table 2:** Comparison of modeling accuracy of six feature extraction methods.

Feature Extraction Method	R^2^	RMSE (μg/L)	MAE (μg/L)
Correlation coefficient method	0.5031	13.9213	10.7663
band ratio method	0.6471	11.7333	9.4349
PCA	0.7581	9.7144	7.3036
SPA	0.5862	12.7042	8.5784
SA	0.7746	9.3762	7.5796
TSSA	0.9654	3.6723	3.1461

## Data Availability

The core code for the local optimization process of the TSSA algorithm can be obtained at https://github.com/peimessi/Local-optimization-of-TSSA-algorithm, accessed on 8 January 2023.
